# Ten simple rules for leveraging virtual interaction to build higher-level learning into bioinformatics short courses

**DOI:** 10.1371/journal.pcbi.1010220

**Published:** 2022-07-28

**Authors:** Wendi Bacon, Alexandra Holinski, Marina Pujol, Meredith Wilmott, Sarah L Morgan, on behalf of the European Molecular Biology Laboratory—European Bioinformatics Institute Training Team

**Affiliations:** 1 School of Life, Health & Chemical Sciences, Faculty of Science, Technology, Engineering & Mathematics, The Open University, Milton Keynes, United Kingdom; 2 European Molecular Biology Laboratory, European Bioinformatics Institute (EMBL-EBI), Wellcome Genome Campus, Hinxton, Cambridgeshire, United Kingdom; Dassault Systemes BIOVIA, UNITED STATES

This is a *PLOS Computational Biology* Methods paper.

## Introduction

The emergence of the Coronavirus Disease 2019 (COVID-19) crisis forced training providers worldwide to move their face-to-face (F2F) courses to virtual environments, challenging course organisers to transfer successful F2F concepts into a virtual format. While recording traditional stand-and-deliver lectures was simple enough, facilitating interaction among course participants proved the key challenge, as virtual environments were new to trainees, trainers, and organisers.

Interaction in learning always carries an element of risk, whether F2F or virtual. Sometimes, groups do not get along, a strong personality takes over a course discussion, or trainees get lost in activities as they do not feel comfortable asking for help. Interaction in a virtual environment amplifies this problem as disengaging is easier. Trainees can leave their camera and mic off, leave meetings, or simply not attend more easily than in a F2F setting. Trainers too can struggle, as they are unable to walk around the room peering at screens, easily monitoring trainee progress, and initiating casual chats. Trainees can then become lost and feel excluded. Creating a comfortable and efficient interactive learning atmosphere that satisfies a wide variety of learning preferences and home/office-working settings is challenging but crucial for an efficient learning experience in a classroom setting [1]. Indeed, well-planned and well-facilitated interactive learning ensures that trainees feel comfortable and included, allowing them to fully engage with each other, the trainers and the training material.

In addition to learning, training courses provide networking opportunities—particularly crucial for early career researchers hoping to find collaborators. This networking can happen organically in a F2F setting during poster sessions, over coffee or during dinner. However, this is not without its challenges—more introverted trainees may feel uncomfortable in these networking situations. Entire books are dedicated to networking for those that dislike networking [2]. Delivering networking virtually requires extensive restructuring—simply leaving nibbles in a seminar room is no longer feasible—but also provides an opportunity to better capture those who feel alienated in F2F networking events.

While virtual interaction is not without its challenges, such activities distinguish virtual courses from online learning. Scientists can now freely access recorded lectures, webinars, practicals, and more. Students in traditional lectures fail to retain even half of the information presented, particularly when lectured with powerpoint [3]. Nevertheless, traditional lectures, even in cutting-edge bioinformatics fields, are common (there are over 60,700 videos on single-cell RNA-seq analysis alone). Unsurprisingly, the demand for training courses in such fields remains despite such free resources [4]. Training courses must have higher value. The unique value of live courses is, therefore, in the interaction.

EMBL-EBI has a long experience in developing, organising, and delivering impactful F2F bioinformatics training courses [5]. The COVID-19 crisis also prompted a rapid move to virtualise our training courses. We have run over 26 virtual training courses from our core team for up to 30 participants each so far, testing, assessing, and refining a variety of interactive strategies. Now, we present 10 simple rules that will create interactivity in a virtual training course to develop community and improve learning.

## Introducing the rules

**Fig 1:**
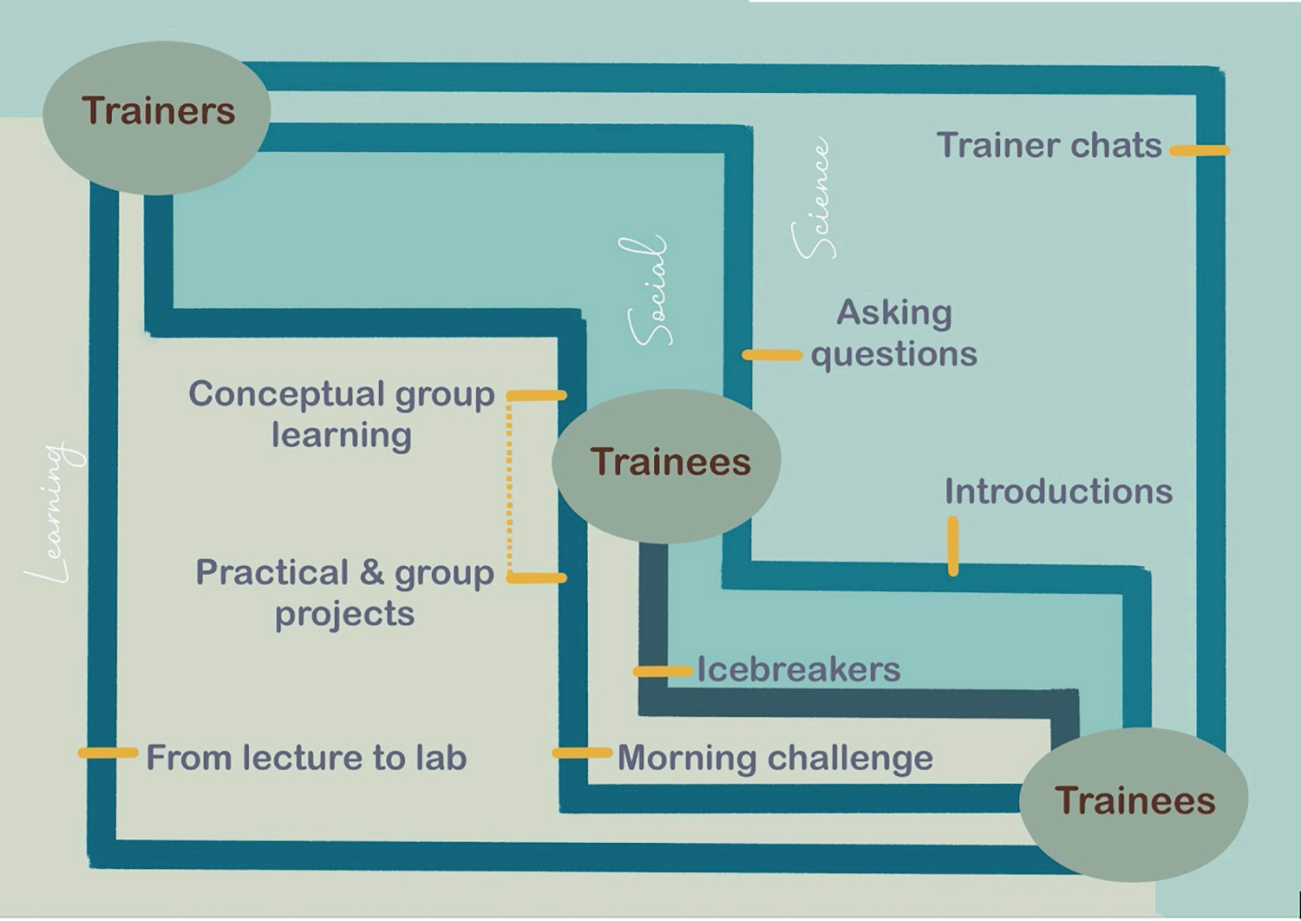
Interpersonal and intertopic interactions necessary for high-impact learning.

A wide variety of interactions are crucial for setting up a successful learning environment (Fig 1). Interactions between trainees as well as between trainees and trainers are key for group and directed learning. Such interactions are facilitated by recognising and structuring social, scientific, and learning activities.

In order to accomplish this plethora of interactions across topic and person, we present our top 10 rules (summarised in S1 Table). The first 5 rules introduce a framework that provides room for social and scientific interaction that allows for the development of a networking and learning community. Having built this inclusive community, Rules 6 to 10 use virtual interaction to deepen learning in bioinformatics concepts and analysis.

Given the wide array of technology to facilitate interaction and variation in terms, we include an overview of the terminology used throughout the text (Table 1). In terms of choosing a specific platform, consider the ease of use for trainees; ease of use and access for trainers to populate their session information and directions; and ease of alteration for after a course, should institutions wish to make the materials publicly available after the course. Many platforms allow for a variety of activities and icebreakers, from Bingo, Name that song, and Draw and guess to quizzes. The training organisers must consider what suits the course—and test this in advance. Consider the following:

- number of participants (some platforms or applications have limits);
- type of participants (do they have a similar background? Will anyone feel left out by a given activity, i.e., agism across songs, etc.?);
- time in the programme for social/networking activities (evening drinks often exclude those with caring responsibilities, therefore networking should take place in the day programme); and
- how many activities (i.e., daily, short, long, scientific, and social separate or combined).

**Table 1:** Summary of the commonly used terminology in course organisation used to distinguish participation, sessions, and applications.

Name	Description	Other names and examples
Trainees	People attending the course to learn	Participants, delegates, and students
Trainers	People delivering the training	Speakers
Organisers	People responsible for the logistical planning and running of the course, from selecting trainers, to creating programmes, to communicating with trainees	Event organisers; scientific training officers; scientific organisers
Participants	Trainees, trainers, and organisers	
F2F	In person, face-to-face	Onsite, live
Virtual	Online	Remote
Teleconferencing	Live platforms used to deliver courses	Zoom, Google Meet, Microsoft Teams, Slack call, and GoToMeeting
Spatial teleconferencing	A subcategory of teleconferencing, where participants can move their avatars around to talk with one another	Wonder, Gather town, and Spatial chat
Messaging application	Text platforms used to communicate	Chat forums Examples: Slack, Zoom chat, and WhatsApp
Channels	Separated threads of communication within the same application	Channels within Slack and Microsoft Teams
Living document	A collaborative document which users can edit simultaneously	Google Docs and Office365
Practical session	A session wherein trainees are given an instruction sheet with a step by step description of how to perform a given analysis, which they proceed to follow	Practicals in groups
Gaming application	Icebreaker platforms used for networking during the course to encourage conversation and discussions	Slido, Mentimeter, Kahoot, and bingo

### Rule 1: Structure, structure, and structure some more!

#### Why?

Structure is critical—so that trainees and trainers can easily navigate the course and have clear parameters for how to interact, where such social norms do not exist in a virtual environment.

#### How did we do it?

Key to structuring our virtual courses was the course handbook, which is a much more detailed version of the programme and collaborative note-taking living documents we previously used in F2F courses. The course handbook is a single location for all resource links, session instructions on expectations and how to participate, and biographical information on trainers and trainees. The handbook is an essential platform that helps trainees navigate through the virtual course and facilitates virtual interaction among trainees and trainers.

We have tested a variety of technologies for the course handbooks (living documents, Google sites, current: Wordpress). Notably, the Galaxy Project Training Network posts their course handbooks on Github (Galaxy Smorgasboard [6]). Our team held course run-throughs with the trainers to ensure that they were aware of how each session would run. This trainer–training information was collated into a living document they all accessed. We piloted weekly training surgeries, where trainers joined to ask questions about their session or troubleshoot ideas. Finally, a lead organising host was nominated for chairing the course, ensuring someone always knew who should be talking.

#### How did it go?

“*Everything we needed was neatly organised into the course handbook. I really liked that there was an overview of the programme timings as well as individual pages for each day*. *It was also very useful to have everything in one place and I particularly like that all the recordings from each day [were] uploaded accordingly after the course. It makes it very easy for us to go through and revise any material we may need more time to look at.*”

For trainer training, course run-throughs and trainer living documents are standard practice across all courses, as this has proven critical to smooth virtual interactions. Training surgeries often had low attendance, but those trainers that did attend found the surgeries invaluable, particularly for newer trainers. Active course hosting has also proven critical. While F2F courses need more hands-off hosting to allow for organic conversation and discussion, virtual courses necessitate more control, as social norms do not yet exist for virtual interaction. We have found that active hosting, such as clarifying when a trainer stops speaking, “Are they to do their practical now? And when will we be meeting back to discuss?,” or “Are they working in groups now, or are they asking questions to discuss with everyone?” can head off a variety of awkward silences or group confusion. Keeping participants verbally informed when things go wrong, for example, if a trainer has not shown up due to a family emergency or internet failing, makes it easier to swap sessions around or change timings without losing the group.

#### Our further recommendations

Key information in a course handbook should include the following:

- course programme and instructions on how the sessions will run (i.e., which platforms to install, session format, etc.), including how to ask questions;
- session links and materials (slide-decks, answer keys, relevant papers, and links to walkthroughs); and
- trainee and trainer background to foster a community.

Successful hosts should

- introduce how questions will be asked and interactions organised;
- provide discussion during silences and encourage discussion from all participants;
- keep to time and topic; and
- communicate technical or course changes (such as times changing or trainers needing to leave).

### Rule 2: Think inclusivity

#### Why?

Just as virtual conferences have increased accessibility and inclusivity, so too has the virtualisation of short courses [7]. Virtual courses cost less in terms of flights, visas, hotels, and time, increasing accessibility across socioeconomic and caring responsibility differences. Virtual courses also provide ample opportunities for increasing inclusivity through democratising interaction, no longer the purview of the highly privileged, extroverted, participative, and available in the evenings for happy hour. That said, the pandemic also meant increased childcare and schooling responsibilities fell on participants, making time management all the more critical.

#### How did we do it?

To take into account those participants with caring responsibilities as well as to alleviate Zoom fatigue, we mixed live with asynchronous sessions, ensuring to record relevant sessions for viewing afterwards. Links to activities completed during live sessions were also made available for asynchronous review or for those unable to attend the live sessions. To support those with hearing disabilities, or, indeed, non-native English speakers where teleconferencing impedes language understanding, live transcript captioning was offered. We included a variety of means for questioning (see Rule 4) to allow anonymity, and together with clear structure (Rule 1) to all interaction, we ensured general comfort and low pressure. A code of conduct was included in our introductory presentation to ensure everyone was aware of appropriate, inclusive behaviour. Course trainers included pronouns in their teleconference and chat forum names, directing participants to do the same. Finally, we added a question to our post-course survey to assess inclusion.

#### How did it go?

While only 28% of participants used captioning, those that did found it beneficial.

“*I struggle keeping up and hearing the correct words, especially when switching between different accents, and although the automated captions don’t cope very well with the unusual words we use in science*, *it does hugely help string the context of a sentence together.*”

The structure (Rule 1), multiple methods for questioning (Rule 4), code of conduct, captioning, and balanced live versus asynchronicity of the programme created an inclusive environment.

“*And since you obviously care about inclusivity (I noticed the pronouns in the Zoom names and emails)…My compliments on also being very autism-friendly. Asking questions via the google doc / in written form—Clear schedule that was actually followed (that’s rare)*—*Being OK with people sometimes having camera’s off—Recordings help a lot, in some cases I left a bit earlier to take a longer break because I was getting too overwhelmed but I still could participate 100%.*”

#### Our further recommendations

- As we are based in the United Kingdom and a large number of our attendees are made up from Europeans, we decided to keep our courses in GMT/BST. Other courses or conferences that span more time zones have recruited trainers from across time zones and offered multiple rounds of the same sessions [6].
- Live captioning struggles with technical language. Prerecorded session captions can be edited to improve this, but does require increased organisation and time.

### Rule 3: Carefully construct introductions and “icebreakers”

#### Why?

Participants that feel comfortable form a proactive learning community that benefits from group learning. Virtual courses must create relaxed social interactivity and community where audio is controlled (i.e., all must be muted to hear someone speak), body language is impossible to see, and structure is crucial (see Rule 1). To combat the reputation of (poorly planned) awkward, lengthy, public and much dreaded “icebreakers,” we must vastly increase the value of such introductory sessions. Finding common ground facilitates networking, learning, and interaction, ensuring that participants feel connected to the group and combating the isolation of sitting at a computer alone.

#### How did we do it?

We created various levels of introduction and icebreakers from pure social to scientific. First, introductory virtual icebreakers set up an informal environment. Breakout groups of up to 5 trainees had 3 to 5 minutes to complete a specific task (i.e., introduce themselves and find someone who has a cat, etc.). By performing this task 4 to 5 times, each trainee met most of the 30 trainees without having to present themselves to 30 people at once. Later, we transitioned into more scientific networking with lightning talks—a variation on poster sessions. Our team tested a variety of lightning talk formats: live versus prerecorded; from 90 seconds to 3 minutes; with spoken questions or typed questions in a living document. All talks started with a “personal,” nonscience introduction, followed by their research overview.

In more formal courses aimed at specifically principal investigator career levels—where networking is more standard—participants were divided into pairs to present themselves, after which they presented their partner to the whole group. To maintain the conversational environment, we started each day with short activities with an element of introduction but delving into the learning objectives (see morning challenges, Rule 6).

#### How did it go?

“*Really enjoyed the games and the flash talks were really excellent*. *Lots of lively discussion and questions—very helpful.*”

Introductions were popular, with multiple mentions in the course feedback. Importantly, trainees did not leave part-way through. Lightning talks varied in reception. Live lightning talks often went too long, with difficulties in slide progression. Some talks received many questions, while others received awkward silence. Finally, clear differences across levels of English speaking ability when nervous were prominent. On the other hand, prerecorded lightning talks were clearer, more confident, and kept to time. By having an asynchronous questions document for lightning talk presenters, participants could rewind and relisten to talks, making it easier to add questions and discuss throughout the course. Many participants even directly messaged each other for live chat discussion. Trainers could also easily add questions to less popular lightning talks, ensuring a sense of inclusion in the course. Finally, shorter talks functioned better. For example, for 30 participants at 3 minutes of talks each, equaled 90 minutes of talks. Even for shorter sessions of 90 seconds each (45 minutes total), we spread lightning talks across 3 sessions, each leading into a break.

#### Our further recommendations

- The order of interaction is key—social icebreakers start a course, before evolving into scientific networking and finally group learning.
- We recommend prerecorded lightning talks for scientific networking that encourages inclusivity.

### Rule 4: Manage questions

#### Why?

Questioning is a fundamental tool for science education [8], yet questioning can be difficult to manage in a large group. Virtual settings provide opportunities to overcome many of these issues, but can also overwhelm participants faced with tracking multiple forums, raised hands, and verbal questions. Structure is crucial to prevent dilution of important messages, to ensure that questions are directed to the right people, and to prioritise questions appropriately—i.e., connection problems or practical questions need quick answers, while conceptual questions can be answered anytime.

#### How did we do it?

**Fig 2:**
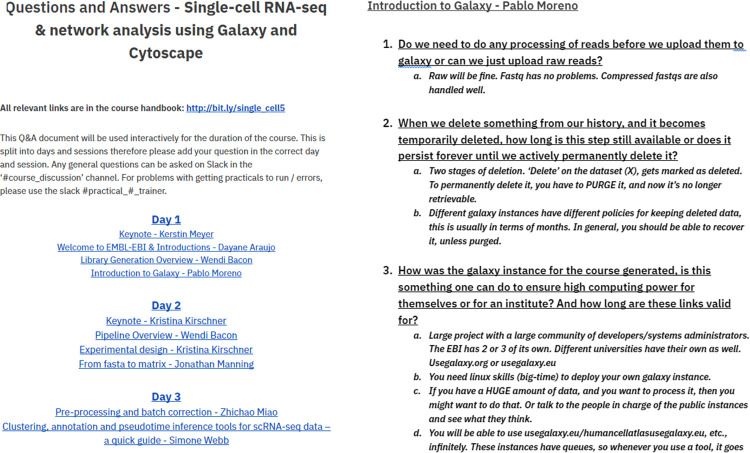
Question and answer document from the *Single-cell RNA-seq & network analysis using Galaxy and Cytoscape course 2021*.

Traditional lectures were prerecorded to allow for asynchronous study. We included extra time in the programme to watch these prerecordings and made them available 2 weeks prior to the start of the course. Trainees posted questions either anonymously or with their name on a living questions and answers (Q&A) document (Fig 2), which was read out by the session host to the trainer during live Q&A sessions, which were recorded. Trainees could also raise their hands to clarify or follow-up on answers themselves, in addition to adding questions to the Q&A document. Trainers often brought teammates to take notes on the Q&A document.

For activities and practicals, separate chat forums were used, for example “#practical_1” or “#morning_challenges.” This ensured that trainers were not notified of other trainers’ discussions. If a trainee was stuck on a practical, they could either scroll through previous threads for solutions or post a question, which was answered quickly during monitored hours. We also included a #technical_support channel monitored continuously by our systems administrator and course organisers for any issues with teleconference connections or navigating the course. Such problems require quick answers but should not notify other trainees. Trainers created threads for each question and added an emoji checkbox on the original question when answered, for easy organisation among themselves as questions appeared. Trainers additionally provided support within the live teleconferencing room.

#### How did it go?

Prerecorded lectures were popular and often led to over 30 questions during a Q&A session—far higher than in a traditional F2F lecture or even in a live virtual lecture. As participants populated the Q&A document in advance, trainers appreciated seeing the questions beforehand, and in some cases arrived with extra slides to address them. The often anonymous Q&A document, coupled with structured chairing (i.e., host asking questions and checking hands), also discouraged commentary-style questions that often eat up time and do not engage the group.

“*… if I didn’t catch something during the lecture*, *I could just rewind and listen to it again.”*

“*I think the use of the [living document] for questions on talks was also really beneficial*. *I think especially in the beginning, it helped me ask questions I felt a little less confident asking out loud.*”

#### Our further recommendations

- Live sessions should contain interaction, whereas traditional, noninteractive sessions should be recorded. Such asynchronicity has advantages, such as listening at various speeds, or pausing and rewinding as needed.
- We recommend checking the Q&A document periodically as questions appear and have a cut-off of when trainers will no longer check.
- We disable the live chat during Q&A sessions to ensure any typed text is captured on the Q&A document, thus preventing trainers from having to check multiple locations for questions and discussion.

### Rule 5: Instigate trainer chats

#### Why?

Networking between trainees and trainers is a key value that distinguishes live courses from YouTube seminars. In a F2F setting, such networking opportunities take place in a lunch or coffee break, which is difficult to facilitate in a virtual course where breaks are used to combat “Zoom fatigue” [9].

#### How did we do it?

In addition to Q&A sessions focusing on a specific lecture, our team included trainer-trainee casual chat sessions. We piloted 3 types of trainer-trainee interactivities: “Talk with the Trainers,” “Bring your own data,” and “eMeet the trainers.”

In “Talk with the Trainers” sessions, trainers visited breakout rooms of up to 5 trainees to chat. In the event of a quiet room, trainers were prepared to share their experience on a research obstacle they overcame. Trainers rotated rooms every 15 minutes, for a total of 6 rotations. Following feedback, our next iteration included a 15-minute break in the middle.

The “Bring your own data” workshops were run for EMBL PhDs students. We split the content into modules. At the end of many modules, a “Bring your own data” workshop was offered, described as a “surgery” or “knowledge exchange” session. Up to 26 PhD students attended these sessions to discuss questions specific to their research projects. Students could show their own data to the trainers. Trainers sent surveys to their trainees ahead of the course to learn about their interests and to collect questions to discuss.

In “eMeet the Trainers,” we invited the trainers and trainees to join a Spatial teleconference to speak with one another. In these spaces, participants could navigate their avatars around to join or leave chats and could also lock conversations to make them private.

#### How did it go?

Trainees often rated such direct interaction with trainers as the best part of the courses. Trainers appreciated the interaction and the opportunity to offer high-impact sessions with minimal preparation (in contrast to building a lecture).

“*I felt that the multiple channels*—*Slack, Wonder, etc.—gave us the opportunity to interact with other delegates, trainers, and keynote speakers effectively … I thought the networking aspects were quite good …*”

#### Our further recommendations

- For “Talk with the Trainers,” clear session explanations and rotation countdowns (or a teleconferencing service that asks permission before moving someone between breakout rooms) ensure smooth transitions between discussion groups.
- For “Bring your own data,” several trainers attended to guarantee good discussion. Conversely, one experienced trainer who feels comfortable with the format can also drive discussion.
- For “eMeet the Trainers,” a course organiser stayed in the main virtual teleconference room to help any participants that experienced technical issues in switching platforms.

### Rule 6: Begin each morning with a challenge

#### Why?

Comfort with interaction in a virtual environment must be maintained throughout a course, necessitating introductory, social interactions to begin each day. However, if trainees find that the mornings are purely social interaction, many will start skipping them for extra sleep—we must ensure value for time, having already set up a welcoming and participatory virtual environment. Therefore, we now begin describing rules where interaction elevates learning outcomes. A key area for improving learning outcomes in bioinformatics is troubleshooting.

Troubleshooting is a key component of science—bench or computer-based!—but during practicals, errors take on a different meaning. Because practicals are curated code that anyone should be able to run, getting an error message can be disheartening and leave scientists isolated, afraid to ask for help. Reframing these errors as group learning experiences helps combat the frustration learning curve in bioinformatics and ultimately build resilience and patience—a key component for learning to code [10].

#### How did we do it?

**Fig 3:**
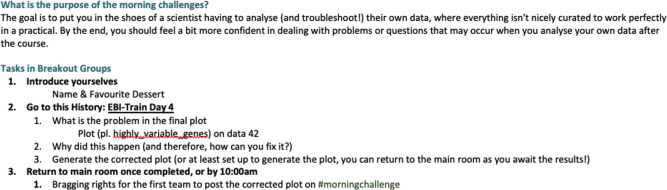
Example of a morning challenge instruction sheet.

We started each day with a 15-minute “morning challenge (Fig 3)”, wherein trainees were randomly distributed into groups of 4 to 6 and given instructions to introduce themselves, share social information (i.e., favourite food), and then rapidly move on to troubleshoot an error or diagnose dodgy data. Trainers often used this challenge to choose common errors that occur in the upcoming or previous practical, so as to prevent future issues or resolve unspoken errors in the past. This isolated, controlled, troubleshooting “challenge” was a stepping stone from curated practicals to coding in the lab.

#### How did it go?

Morning challenges encouraged peer-to-peer support, reframing troubleshooting as an exciting challenge rather than a sign of failure. Socially, placing the challenge prior to a live Q&A led to more questions and lively discussion. Following a challenge with a practical led to improved engagement with chat forums and peer-to-peer assistance. The challenges were popular confidence-builders, often rated the best part of the course.

“*…the troubleshooting was very good*, *because that is the most likely scenario that we are gonna face when we are on our own, so that helped a lot to learn how to tackle these situations.*”

#### Our further recommendations

- Such short challenges do not require preamble, as trainees were able to follow instructions in their breakout rooms easily.
- By providing answer keys and a group walkthrough of the ‘answer’ at the end, we reinforce the learning.

### Rule 7: Empower learning from lecture to lab

#### Why?

A key obstacle in bioinformatics training, F2F or virtual, is ensuring that trainees can repeat their analysis in their own laboratory environments, far from the carefully curated compute systems of a traditional training course. Course organisers, including EMBL-EBI, have attempted various forms of “analyse your own data” or “bring your own laptop,” but feedback is mixed from trainees (who, short of having significant 1–1 time with a trainer, often struggle) and poor from trainers (who get frustrated dealing with massive data uploads, unfamiliar formats, and differently configured computers from multiple trainees at once). Carefully structured interaction is crucial to achieve this learning objective.

#### How did we do it?

We piloted multiple solutions to ensure application in research labs, ranging from empowering trainees to communicate their needs to systems administrators in their home environments, to selection of publicly available platforms. For developing bioinformatics communication skills, we have ranged from traditional lectures with Q&As, lectures with discussion elements, to “Ask what you’re afraid to ask” chat forums and live sessions with anonymous living Q&A documents. In our Galaxy-based courses, trainees simply accessed public Galaxy through their laptops, just as they would for analysing their own data.

#### How did it go?

For empowering communication and infrastructure basis, feedback varied. It is challenging to generate enthusiasm for computer science among biologists. Anonymous questioning was popular and much-appreciated, as many of these questions might be asked in hushed tones over a coffee in a F2F course. The use of publicly available platforms proved, unsurprisingly, the most successful, as trainees often applied workflows to their own data during the evenings of the course, sharing any error codes or analytical tips with each other via chat forums.

#### Our further recommendations

- The learning objective for “lecture to lab” is to identify infrastructure needs to communicate this to home departments in order to set up to apply analyses. This is difficult to achieve. We ultimately recommend circumventing this need as much as possible. For example, you can use the Galaxy Project (or similar cloud-based accessible analysis).
- Note: We recommend precourse virtual machine installation drop-in sessions to ensure participants are prepared when the course starts.

### Rule 8: Transform lectures into conceptual group learning

#### Why?

Theoretical learning is necessary for trainees to complete practicals. Having employed a wide variety of tools to create a welcoming, inclusive and interactive cohort of trainees, the course is ripe for group learning activities that move beyond traditional lectures.

#### How did we do it?

**Fig 4:**
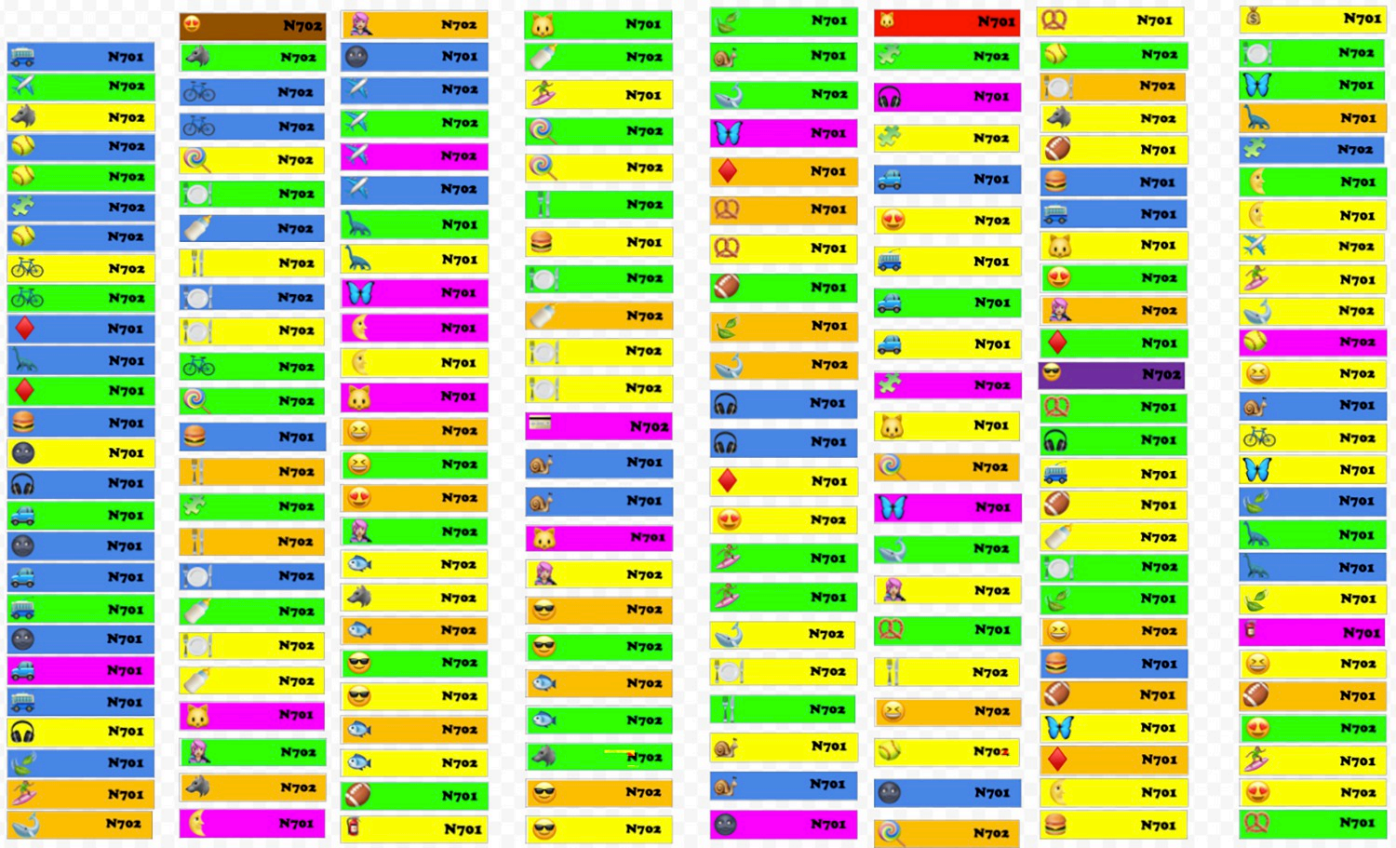
Starting screen of “Reads” in conceptual group learning activity.

We used conceptual group learning for the workflow of single cell RNA-seq analysis, using collaborative living drawings documents and breakout rooms of 4 to 6 trainees. After a short introduction of the activity purpose and learning outcomes, trainees were given a packet of instructions and fake “sequencing reads” (Fig 4). Trainees completed the workflow by physically organising the “reads,” following the analysis instructions. Trainers could keep track of groups by monitoring the living documents. Final figures were compared across groups, leading to a survey on the challenges in analysis, a highly interactive whole group discussion, and a wrap-up presentation.

#### How did it go?

“*I thought the workshop where we had to use Google Docs to "work" through the data was really smart as it helped me understand/visualise why we were doing certain things- sometimes I find it hard when it is just a line of code/a command.*”

For trainers, such activities are easily reproducible across courses and easy to monitor—this activity had one trainer for 30 trainees. The activity was self-contained, which allowed the trainer to rotate through groups to have meaningful 2-way discussion rather than 1-way lecturing. While initial creation of the activity was time-consuming, repeating the activity was easier than repeating a lecture.

#### Our further recommendations

- The activity should be the learning, rather than an assessment. The activity ideally should be self-contained and not require a lecture beforehand.
- Simplicity was appreciated—traditional lectures can pack in excessive content [11].
- Tactical scheduling is key—group work sets participants up to interact more openly; therefore, it is ideal earlier on. From experience, if placed at the end of a course containing an otherwise traditional suite of lectures, trainees are less prepared for such interaction and will give lower feedback.

### Rule 9: Divide into groups for hands-on practicals

#### Why?

Hands-on practicals are vital for bioinformatics training, as lectures alone will not train skills. Practicals are an effective way to enhance trainees’ motivation and extend their knowledge in understanding [12]. However, in virtual environments, trainers cannot easily stand behind computers to assess how a class is doing, or take over a keyboard to get a participant back on track. Trainees are more easily lost, particularly amid the pressure to keep up, just as they may be more easily bored by an overly simple practical. While working in groups is common in F2F practicals, the ease of losing trainees as well as tuning out makes such group work difficult to enact virtually. Yet, working in groups allows trainees to help each other, thereby reducing pressure on trainers to provide 1–1 support for every trainee; to support each other through frustrating troubleshooting; and to develop camaraderie, allowing for easier and more supportive discussions that prevent imposter syndrome.

#### How did we do it?

We piloted multiple versions of practical formats to maintain motivation and encourage peer-to-peer learning, as it was not feasible to have one trainer for each trainee. First, in “Guided practicals,” participants remained in the main room as the trainer and trainees worked through the practical live, raising questions as and when verbally. Second, “Optional breakout rooms” were created, and trainees thus had the additional option to work in a group, choosing their own breakout rooms. In “Random trainer guided breakout rooms,” trainees were randomly assigned to rooms and given a trainer who either stayed with them through the practical or rotated through groups, thus allowing both group work and trainer support in a smaller setting. Finally, in “Level-specific trainer guided breakout rooms,” groups were created based on prepolled level of programming knowledge, thus keeping similar levels of trainees together and better encouraging questions. In all cases, we encouraged trainers to create practical walkthrough videos in advance. This avoided a variety of where-is-the-button questions that are difficult to answer virtually and also ensured that those attending asynchronously or requiring more time after a practical could still perform it.

#### How did it go?

Practical chat forums were active, and in many cases, trainees would directly message trainers for assistance, indicating concern about public questioning. “Guided practicals” without breakout rooms were the least effective, with many participants disengaging. “Optional breakout rooms” were difficult to initially generate trainee engagement, while “Random trainer guided breakout rooms” occasionally led to a silent group as trainees simply completed the practical on their own. “Level-specific trainer guided breakout rooms” were the best from both trainer and trainee perspectives.

“*The PRACTICALS in breakout rooms [were the best part of the course]*. *It was a great idea to divide people according to their level of understanding. This worked well since I feel frustrated when I get stuck on the first exercise and someone else is finished and busy taking coffee. The group I was placed in was good since we had similar challenges and I would often get the solution by listening to their problems/solutions. I would recommend you split people in similar groups for future courses.*”

Walkthrough videos were also highly praised.

“*The galaxy tutorials were good, but the*…*recorded demonstration was very very helpful. Several times I didn’t quite follow from the instructions what I was meant to do, and could quickly switch to the demo to see.*”

#### Our further recommendations

- Practical walkthrough videos could prevent many problems and keep trainees engaged.
- It was important to distinguish between conceptual questions (i.e., useful in a Q&A document for conceptual learning) versus practical problems which need rapid answers from trainers.

### Rule 10: Finish courses with practical group projects

#### Why?

Practical group projects are an active teaching strategy that allow participants to collaboratively analyse biological data and solve biologically relevant questions through interaction and communication with other group members over a couple of days. Projects allow trainees to put into practice everything they have learned in the course, by providing trainees with a realistic biological/bioinformatics use-case, dataset and some suggested aims to work towards. It is the responsibility of the group to independently develop additional realistic and reasonable research aims and work out a joint work plan to reach them, while being guided by mentors, skilled bioinformaticians. The group produces a joint end-product, for example, a slide presentation, to present their results. Group projects have been included in bioinformatics F2F courses and have improved the student’s learning experience [13].

#### How did we do it?

We have piloted multiple group-based projects in virtual courses. Projects spanned between 2 or 3 days. Project groups comprised 5 to 6 trainees in breakout rooms. Trainers monitored chat forums set up specifically for the group work, allowing participants to call for help as needed. For the group project, we aimed to keep the same level of independence and self-organisation as in the F2F courses; however, we were also aware that trainees might collaborate less efficiently with each other, potentially losing trainees in the process. Trainers therefore provided more granular instructions and aims for the project work compared to the F2F course, as well as a realistic timeline and milestones as orientation for the trainees. These plans allowed for flexibility and were shaped by the trainees in agreement with the trainers. To ensure interaction and collaboration within the groups, trainers and trainees also agreed on regular “check-in” times to discuss the current work progress and mutually agree on next steps. In some cases, the groups split into sub-groups of 2 to 3 people. These small groups allowed all trainees to get actively involved in the data analysis. Trainees were provided Lab Notebooks (living documents) to take collaborative records of the work progress as had been done in previous F2F courses [13].

Uniquely in the virtual course, group projects culminated in a presentation delivered to the whole course, providing the opportunity for course-wide discussion and reflection on achievements and challenges. In one course, each trainee in a group presented in a breakout room with a member of each other group. In this way, every trainee had to both present and answer questions, which ensured equal distribution of work and understanding across the participants.

#### How did it go?

We observed that trainees were more inclined to have their cameras on when working in smaller groups in breakout rooms than in an interactive lecture in the full group.

“*I think [it’s] difficult to try [to] foster the same team working then online but I felt once within our groups people talked more.*”

However, trainers also reported that they sometimes struggled to encourage group members to effectively collaborate with each other. Regular live “check-ins,” and the formation of sub-groups can help to drive interaction, and the trainer—in a virtual course even more than in a F2F course—not only has the role of a scientific mentor, but of a social one who facilitates virtual collaboration and interaction.

“*I enjoyed very much also the group project*, *I was able to see how the other delegates approach the data (what they consider and why they proceed in a certain way).*”

#### Our further recommendations

- Group projects should run near the end of a course, after trainees and trainers have developed a convivial atmosphere.
- Ideally, trainers should provide clearly formulated exercises and should discuss with the group a realistic time frame by when certain steps can be reached.
- The group projects should ideally start with short scientific and social icebreakers to give the group and the trainers the chance to get to know each other. This helps to create a comfortable and more engaging atmosphere

## Discussion

Our 10 simple rules summarise over a year’s worth of virtual course delivery as the result of the COVID-19 pandemic. We converted multiple layers of interaction found in F2F courses into virtual counterparts, be it across people (trainees to trainees or trainers) as well as topic (social, learning, and networking) (Fig 1). Ensuring these interactions are clearly structured and purposeful is vital to battle “Zoom fatigue” [9] and dropout and ultimately to distinguish virtual courses from freely available video lectures and resources easily found online. Here, we summarise common problems in course delivery and how they are solved by these simple rules (Table 2). We additionally identify how creating an interactive environment deepens learning (Table 3).

**Table 2:** Common problems in virtual course environments and how the 10 simple rules can fix them.

Challenge	Common problems	Rules	Ideal outcome
Awkward silence—logistics	Trainees unsure when to speak (during the talk? after?) Trainees unsure how to speak (chat, raise hands, etc.)	1	Trainees clearly understand expectations and format, thereby engaging fully with session
Awkward silence—social	Icebreakers are awkward and forced Trainees stop attending interactive sessions Interactivity resets each morning	3 and 6	A chatty and supportive learning community of trainees feel comfortable asking each other their interests and sharing their passions
Feeling alienated—logistic	Trainees unsure where to be, arrive late and/or miss sessions, feel alienated or excluded	1	Trainees easily navigate a course, never feeling anxiety about missing out
Feeling alienated—social	Trainees feel culturally or societally unwelcome	2	All trainees feel welcome
Feeling alienated—imposter syndrome	Nobody asks questions during someone’s lightning talk Trainees feel afraid to ask questions and that they might look foolish. Trainees are afraid to approach the trainer	3, 4, and 5	Trainees feel comfortable discussing ideas, questions, or interests with other trainees and trainers
Information lost in chat forums	Chat forums are busy and overwhelming, burying information and leading to repeated or missed questions, notifying and overwhelming the wrong trainers. Solve-me-now issues not seen until too late.	1, 4, 9, and 10	Information is clearly prioritised to ensure rapid answers where needed. Trainees can support one another and easily find previous solutions. Forums are lively but manageable
Interaction for interaction sake	Icebreakers are awkward and forced	3, 6, 8, 9, and 10	Interaction has a clear purpose, to support a learning community or learning
Dominant personalities take over discussion	Overly specific “solve my research problem” questions dominate time, alienating other trainees. Posturing questioning to appear smart dominates time and alienates other trainees	4, 8, 9, and 10	Discussion is democratic, with many voices taking part both live and on forums in informal, supportive, and inclusive communication. All trainees have their questions answered.
Trainees never finish the practical or project	Trainees become frustrated with errors, are afraid to be wrong, and afraid to ask for help (see alienated above)	6, 9, and 10	Trainees recognise that troubleshooting is normal, and are happy to ask for help and share solutions
Trainers overwhelmed	Trainees bring “Solve my research” questions. Trainees are not all able to access trainer equally (see dominant personalities above). Some trainees become bored, waiting for practicals to move on, or for trainers to answer questions. Trainers overwhelmed by questions, feel expectation to individually walk each trainee through steps. Unexpected questions fluster a trainer, causing them to lose the thread of their talk and the audience to lose focus	4, 7, and 9	Questions are appropriately organised, allowing trainers to prioritise what needs answering immediately, as well as what is beyond the scope of the course. Trainees can move through workflows at their own pace, with trainers or fellow trainees easily accessible in case they get stuck.

**Table 3:** Common learning objectives in virtual course environments and how interaction elevates them.

Learning content	Level often given without interaction	Rule	Level given with interaction
Troubleshooting	Repeat (Level 1)	6	Solve (Level 3)
How to start analysis at home lab	None	7	Define (Level 1) or execute the analysis on data of choice (Level 3) with public platforms
Algorithms—what does this button do?	Repeat (Level 1)	8	Interpret (Level 3)
Analysis skills	Repeat the analysis (Level 1)	9	Discuss and explain (Level 2)
Analysis skills	Repeat the analysis (Level 1)	10	Execute the analysis on data of choice (Level 3)
Parameter choice	Repeat the analysis (Level 1)	10	Question, test, compare, and contrast (Level 4)

Overall, planning a virtual course takes time and resources. There are not always direct corollaries from F2F to virtual or from virtual to F2F. For example, while we could not reproduce networking coffee breaks virtually, as participants needed time away from screens, we could introduce novel sessions exploiting breakout room functionality, such as “Talk with the Trainers” breakouts or introductory games. On the other hand, such virtual breakout activities would be difficult to reproduce in a F2F environment limited by noise and space.

By focusing on the environment and how we organise interaction, our 10 simple rules provide a unique toolkit to complement what is already known about optimising platforms for training [14] or the scientific content in short courses [15]. Carefully planned interactive elements, indeed, open up new avenues for content—such as focused discussion of individual needs, troubleshooting as its own skill set to learn, or inclusive exploratory projects.

We have found that balancing interaction is critical to creating an interactive virtual environment. As described in our introductions and icebreakers section in particular, interaction for interaction’s sake is not received well. Similarly, the new culture of back-to-back online meetings and “Zoom fatigue” means that interaction, no matter how carefully planned, needs breaks and variety. By mixing prerecorded traditional talks with Q&A forums, problem-solving sessions and interactivities, we can combat such fatigue and maintain an enthusiastic, communicative, and participatory audience.

While we have focused on bioinformatics courses, the general principles of developing interactive communities and driving high levels of learning could apply to any short-course scenario, simply trading the bioinformatics practicals or projects for another applied skill set. In a world shifting to virtualise professional and career development courses previously offered only F2F, our 10 simple rules create a framework for deeper learning. Ultimately, interaction is more than just keeping people awake—it is driving people to implement, organise, argue, and conjecture in a way that traditional, noninteractive and singular learning struggles to allow.

## Conclusions

Our 10 simple rules for leveraging virtual interaction in short bioinformatics courses, developed from our experience of over a year of organising virtual courses at the European Bioinformatics Institute, drive trainees into higher levels of learning and thereby distinguish virtual live courses from currently available online recordings and resources.

## Supporting information

S1 Table Summary of the 10 rules for using virtual interaction to build higher-level learning into bioinformatics short courses. (DOCX)
