# Correction: Ten simple rules for leveraging virtual interaction to build higher-level learning into bioinformatics short courses

**DOI:** 10.1371/journal.pcbi.1010964

**Published:** 2023-03-07

**Authors:** Wendi Bacon, Alexandra Holinski, Marina Pujol, Meredith Wilmott, Sarah L Morgan, on behalf of the European Molecular Biology Laboratory—European Bioinformatics Institute Training Team

The Acknowledgments section contains spelling errors. Please see the correct Acknowledgments below.

We particularly acknowledge Juanita Riveros for creating the figure to demonstrate the types of interaction. We also acknowledge the members of the EMBL-EBI Training Team who delivered courses in 2020 and 2021: Mohamed Alibi, Dayane Rodrigues Araújo, Adam Broadbent, Cath Brooksbank, Patricia Carvajal Lopez, Derrin Crawte, Prakash Singh Gaur, Piraveen Gopalasingam, Thomas Hancocks, Marta Lloret Llinares, Vera Matser, Brett McClintock, Michelle Mendonca, Ajay Mishra, Rebecca Nicholl, Charlotte Pearton, Emily Pomeroy, Anna Swan, Daniel Thomas-Lopez, Ian Willis, Jane Reynolds, Lucie Smith and Deborah Rice.
