# Supplementary material for: Ten simple rules for leveraging virtual interaction to build higher-level learning into bioinformatics short courses
Source: PLoS Comput Biol. 2022 Jul 28;18(7):e1010220. doi: 10.1371/journal.pcbi.1010220 (PMC9333319; doi:10.1371/journal.pcbi.1010220)
Supplement: S1 Table — (DOCX) [file pcbi.1010220.s001.docx]

| **Rules** | Role | Short Description |
| --- | --- | --- |
| Rule 1: Structure, structure, and structure some more! | Community Development | In a virtual course, where trainees can get easily lost and isolated, it is essential to provide a clear structure on where to be, what’s expected, and how to interact. Trainers also need similar support. |
| Rule 2: Think inclusivity | Community Development | Various individual learning preferences require the application of varied training approaches such as: recorded lectures; shared Question and Answers documents allowing anonymity; code of conduct; and live transcript captioning |
| Rule 3: Carefully construct introductions & 'ice-breakers' | Community Development | Introductions and icebreakers will help everyone to get to know each other better, feel more comfortable when interacting with each other and thus form a pro-active learning community. Clear structure and purpose is key to avoiding awkward silences and engaging participants. |
| Rule 4: Manage questions | Community Development | A virtual setting offers many options to ask questions, which can be chaotic. Clear instructions are vital on how, when and where to ask questions. |
| Rule 5: Instigate Trainer Chats | Community Development | While in a F2F course trainer-trainee networking can take place organically over coffee or lunch, this needs to be facilitated in a virtual setting, requiring clear purpose and structure. |
| Rule 6: Begin each morning with a challenge | Learning | Starting each course day with an interactive learning activity creates a comfortable interactive atmosphere and targets learning objectives not easily attained through non-interactive learning, such as troubleshooting. |
| Rule 7: Empower learning from lecture to lab | Learning | Trainees range in background computer knowledge, but each needs bioinformatics communications skills to efficiently transfer course skills to their home lab. Anonymous questioning and carefully structured interactions allow participants to ask questions about basic computational knowledge. |
| Rule 8: Transform lectures into conceptual group learning | Learning | For conceptual group learning, participants were grouped with 4-6 other peers and were provided fake 'sequencing reads' as well as instructions to physically organise the 'reads'. These activities are simple collaborative activities that are self-contained and allow for efficient group learning of otherwise abstract concepts. |
| Rule 9: Divide into groups for hands-on practicals | Learning | Practicals are essential for bioinformatics training courses. In a virtual course, however, a trainee - isolated from other participants - is more likely to get lost. We grouped participants by experience level where they could support each other when working through the exercises. |
| Rule 10: Finish courses with practical group projects | Learning | Practical group projects allow for the collaborative exploratory analysis of data. For virtual practical group projects, we organised participants into breakout rooms to work collaboratively on a realistic biological scenario, with more directed milestones and timelines than in a less structured, F2F environment. |

**Supplemental Table 1: Summary of the ten rules for using virtual interaction to build higher-level learning into bioinformatics short courses**
